# Pelvic Abscess after Cesarean Section Treated with Laparoscopic Drainage

**DOI:** 10.1155/2021/8868608

**Published:** 2021-06-10

**Authors:** Yumi Murayama, Tomohito Tanaka, Hiroshi Maruoka, Atsushi Daimon, Shoko Ueda, Masahide Ohmichi

**Affiliations:** ^1^Department of Obstetrics and Gynecology, Osaka Medical College, Takatsuki, Japan; ^2^Translational Research Program, Osaka Medical College, Takatsuki, Japan

## Abstract

Cesarean section (CS), the rate of which is increasing worldwide, may be associated with complications. Although pelvic abscess after CS is rare, it is difficult to treat. We herein report two cases of pelvic abscess treated laparoscopically after CS. The abscesses of the patients were located in the pouch of Douglas and the uterine scar after CS, respectively. Several days after CS, the patients presented with lower abdominal pain and fever. Laparoscopic drainage was performed because imaging revealed a pelvic abscess that was not amenable to drainage through interventional radiology. The patients recovered from infection and were discharged four days after drainage.

## 1. Introduction

The rate of cesarean delivery is increasing worldwide [1]. Cesarean section (CS) may be associated with both short-term and long-term complications, including bladder injury, scar pregnancy, uterine rupture, and abnormal placentation at subsequent pregnancy [2]. Although surgical site infection after CS is rare, it is difficult to treat. Antibiotics may be first choice of treatment for patients with infection after CS, and if unresponsive, drainage may be needed for patients with abscess [3]. An image-guided drainage is standard therapy; however, it may be difficult to perform in some cases. In those cases, laparotomy drainage has been performed; however, a recent study showed that laparoscopic drainage was associated with a lower rate of postoperative abscess, scar infection, and bowel obstruction in comparison to drainage performed with a laparotomy approach [4]. We herein report two cases of pelvic abscess after CS treated with laparoscopic surgery.

## 2. Case Presentation

This study was approved by the Osaka Medical College Clinical Research Review Board, and the patients gave written informed consent for publication.

### 2.1. Case 1

**Figure 1:**
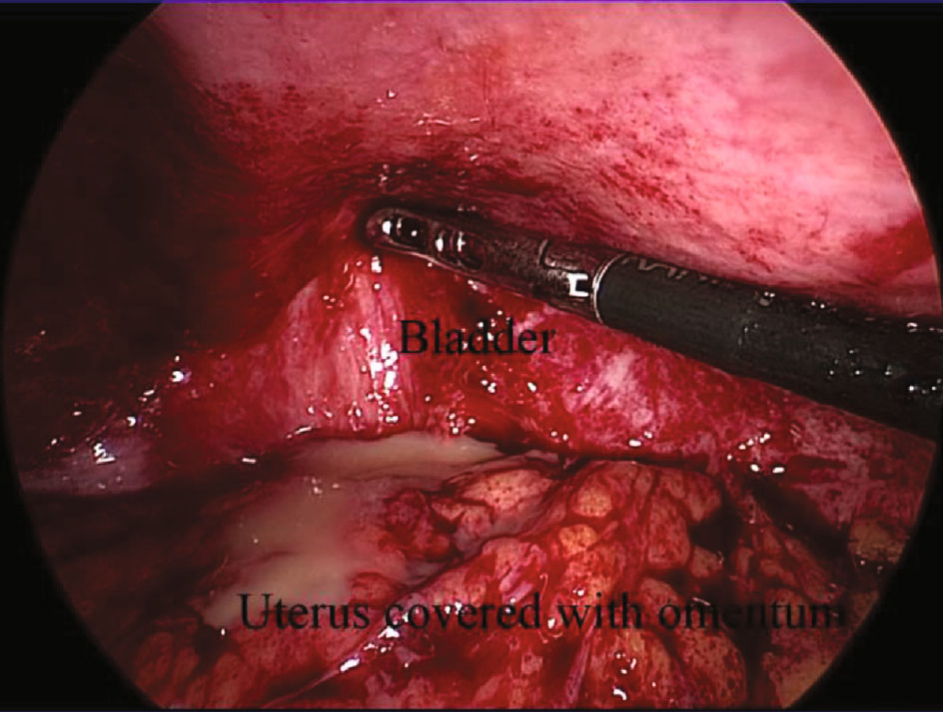
An intraoperative laparoscopic image of the abscess, which was found on the left side of the uterine scar between the posterior bladder wall and the anterior lower uterine body.

A 30-year-old woman (gravida, 2; para, 1) underwent CS due to breech presentation. The patient had no symptoms of infection, including temperature or rupture of membranes before CS, and received prophylactic antibiotics to prevent surgical site infection after CS. Lower segment CS was performed with Pfannenstiel's method. At CS, there were no abnormal findings (e.g., endometriosis, adhesion, ovarian tumor, or staining of amniotic fluid). Double layer closure was performed with 0-PDS to repair the uterine incision. The patient delivered a 3500-gram boy who was in good condition; CS was completed without complications. However, the patient presented with abdominal pain and fever 10 days after CS. Antibiotic therapy was administered (ampicillin (4 g/day) plus gentamicin (160 mg/day) and clindamycin (1200 mg/day)) for 5 days; however, she showed no improvement. A blood examination revealed an elevated white blood cell (WBC) count (12570/*μ*l (normal range, 3300-8600/*μ*l)) and C-reactive protein (CRP; 1.2 mg/dl (normal range, <0.3 mg/dl)). Ultrasonography and CT showed an abscess of 6 cm in diameter on the left side of the uterine incision. An image-guided drainage was considered; however, transabdominal CT-guided drainage could not be performed because the abscess was covered with the bladder and bowel. Transvaginal ultrasonography-guided drainage also seemed to be difficult because the abscess was presented in the anterior wall of the uterus; the uterus and bladder obstructed the puncture. Thus, laparoscopic surgery was performed with pneumoperitoneum under general anesthesia. She underwent multiport (four ports) laparoscopic drainage with the modified Diamond method using an umbilical port for the camera and three working ports in the bilateral lower quadrant and suprapubic region. The uterus, especially around the abscess, was covered with omentum, forming an inflammatory barrier. Adhesiolysis and drainage were performed. An abscess, which contained yellowish pus, was found on the left side of the uterine incision between the posterior bladder wall and anterior lower uterine body (Figure 1). The abscess was completely fenestrated and washed with saline. At the end of laparoscopy, a catheter was placed in the abscess cavity. There were no intra- and postoperative complications, and the postoperative course was uneventful. The patient recovered from infection and was discharged 4 days after surgery. At 22 months after CS, the patient underwent a scheduled CS for subsequent delivery. The patient delivered a baby girl who was in good condition. No serious adhesion was found at CS. The postoperative course was uneventful.

### 2.2. Case 2

**Figure 2:**
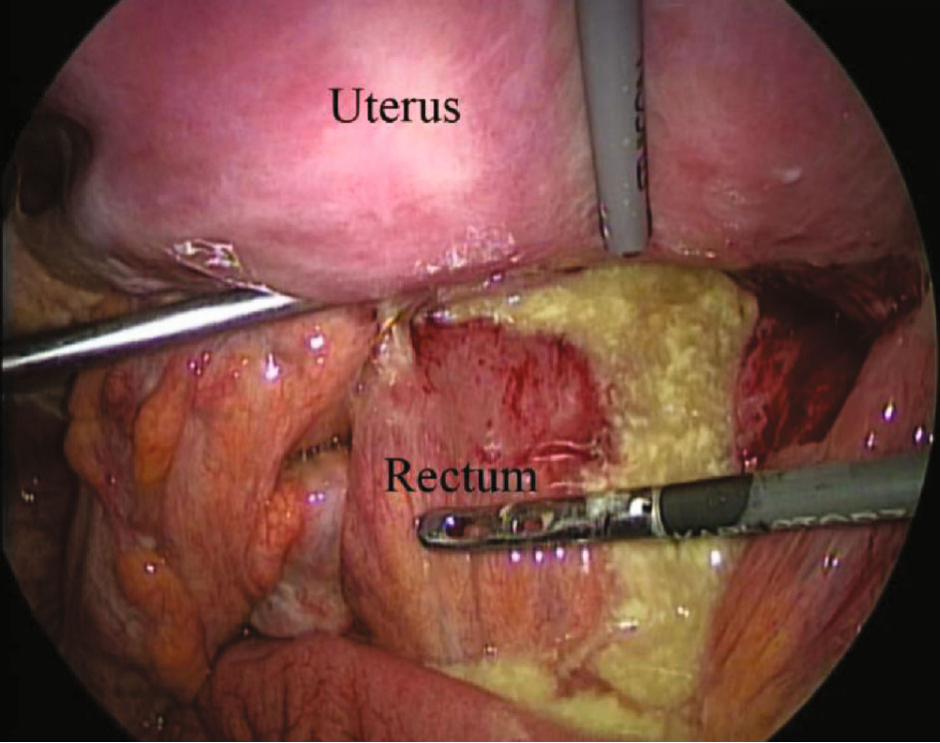
An intraoperative laparoscopic image of the abscess, which was found in the pouch of Douglas.

A 28-year-old woman (gravida, 1; para, 0) underwent CS for the arrest of labor. The patient received antibiotic therapy because of rupture of membranes associated with fever. Lower segment CS was performed with Pfannenstiel's method. At CS, there were no abnormal findings such as endometriosis, adhesion, ovarian tumor, or staining of amniotic fluid. A double layer closure was performed with 0-PDS to repair the uterine incision. The patient delivered a 2850-gram boy who was in good condition. CS was completed without complications. The patient presented with abdominal pain and fever at 6 days after CS. Antibiotic therapy (ampicillin (4 g/day)) was administered for 6 days; however, she showed no improvement. A blood examination revealed an elevated WBC count (914960/*μ*l) and CRP level (24 mg/dl). Ultrasonography and CT showed an abscess of 53 mm in diameter in the pouch of Douglas. An image-guided drainage was considered; however, transabdominal CT-guided drainage could not be performed because the abscess was covered with the bowel. Transvaginal ultrasonography-guided drainage seemed to be difficult to perform because there was not enough space in the abscess for puncture and placement of the catheter. Laparoscopic surgery was performed with pneumoperitoneum under general anesthesia. She underwent multiport (four ports) laparoscopic drainage with the modified Diamond method. The abscess, which contained yellowish pus, was found in the pouch of Douglas (Figure 2). The abscess was completely fenestrated and washed with saline. At the end of laparoscopy, a catheter was placed in the pouch of Douglas. The postoperative course was uneventful, and the patient was discharged on the 4th day after surgery. The patient was free of disease at a 9-month follow-up examination.

## 3. Discussion

In the current report, we presented two cases of pelvic abscess after CS that were treated with laparoscopic drainage. Our report shows that laparoscopic drainage of pelvic abscesses after CS can be an effective approach when an image-guided drainage is impracticable.

CS is currently the most frequently performed abdominal surgical procedure in the world [1]. CS increases the risk of obstetric complications, such as placental abnormalities or uterine rupture in subsequent pregnancies, and is associated with a risk of hemorrhagic complications, urinary and gastrointestinal injuries, wound infection, and myometritis [5]. Although the wound infection rate is usually reported to be around 2%, pelvic organ infection has been estimated to occur in less than 0.2% of cases [6]. Infection after CS usually occurs within the first 30 days after delivery [7]. The risk factors for postcesarean pelvic abscess include younger age, low socioeconomic status, prolonged labor, premature rupture of membranes, multiple vaginal examinations, and cephalopelvic disproportion [8]. The symptoms of pelvic abscess are reported to include lower abdominal pain, fever and chills, nausea, and foul genital bleeding.

Because the clinical symptoms and laboratory findings are nonspecific, ultrasonography and CT/MRI are important for the diagnosis [9]. Pelvic abscesses are classically treated with broad-spectrum antibiotics. This approach fails on occasion, necessitating invasive, or surgical intervention. CT- or ultrasound-guided drainage may be performed as a viable option as it is less invasiveness. It is reported that the successful rate of CT- and ultrasound-guided drainage for pelvic abscess was 83.3% [10] and 92% [11], respectively. In the current cases, it was difficult to perform CT- or ultrasonography-guided drainage because the uterus, bladder, and bowel obstructed the puncture. We also wanted to place the catheter for drainage in the abscess so that we could wash the cavity with saline. For these reasons, laparoscopic surgery was used. An image-guided drainage is standard therapy, and if impracticable, laparoscopic surgery for pelvic abscess after CS is a very useful approach that reduces postoperative pain, the risk of pelvic adhesion, and the duration of hospitalization and affords a good cosmetic appearance [12].

In conclusion, laparoscopic surgery is an effective method for the management of pelvic abscess after CS when an image-guided drainage is impracticable.

## Data Availability

The data used to support the findings of this study are included within the article.
